# The Role of Frontal-Subcortical Circuitry in Neuropsychological Deficit of Attention: Hypothesis and Results in Two Coagulation Disorders

**DOI:** 10.3389/fnhum.2016.00089

**Published:** 2016-03-09

**Authors:** Silvia Riva, Serena Oliveri, Chiara Fioretti, Marianna Masiero, Gabriella Pravettoni

**Affiliations:** ^1^Department of Oncology and Hemato-oncology, University of MilanMilan, Italy; ^2^Applied Research Division for Cognitive and Psychological Science, European Institute of OncologyMilan, Italy

**Keywords:** attention, frontal-subcortical circuitry, coagulation, hemophilia A, thrombotic thrombocytopenic purpura

## Introduction

Coagulation disorders concern a deficiency of the body's functional ability to regulate blood clotting (Peyvandi and Mannucci, 1999). Disorders in thisarea, which may be genetic or acquired, will result in hemostasis-related problems, including different clinical syndromes from easy bleeding or bruising (so-called “hemophilia”) to inappropriate thrombosis (so-called “thrombophilia”; Weisberg, 1996).

Cognitive processes and neurologic disorders have been studied extensively in several disease contexts (Lucchiari et al., 2010; Oliveri et al., 2012; Smorti and Fioretti, 2015; Fioretti and Smorti, 2015). In the field of coagulation disorders, neuropsychological deficits have not been studied extensively, although they represent a frequent occurrence when the diseases involve the central nervous system (CNS) (Riva et al., 2014b, 2015a,b).

In two previous published works of the first author, we studied the neuropsychological deficits in two cohorts of patients affected by rare coagulation disorders with significant impairments, especially in the domain of attention. The two disorders were hemophilia and thrombotic thrombocytopenic purpura (TTP).

## Cohort studies

### Hemophilia

Hemophilia is a rare coagulation disorder in which a crucial blood-clotting factor is missing or deficient (White et al., 2001). Its main treatment is replacement therapy, andis based on intravenous infusions of clotting factor concentrates. These infusions help replace the missing or low clotting factor (Santagostino and Mannucci, 2000).

Clinically, neurological involvement in hemophilia may be determined by endogenous or exogenous factors. In hemophilia, bleeding “can cause peripheral (nerve or plexus) or intracranial (subdural, subarachnoid, intercerebral) lesions” (Weisberg, 1989, p. 2), which may determine neurological complications and functional cognitive disorders. However, neurological complications may be associated with exogenous factors, such as HIV virus infection (Mangiafico et al., 2011). Indeed, a great percentage of adult hemophiliacs (especially those born before 1985) contracted HIV through receiving transfusions of clotting factors drawn from infected blood between 1979 and 1985, when the virus was still unknown (Franchini and Mannucci, 2012). HIV is neurovirulent, and due to direct infection of the CNS, neurological and cognitive disorders may manifest (Blanchette et al., 2002; Becker et al., 2011; Valcour et al., 2011).

In our previous work, we found that patients with hemophilia (especially those who contracted HIV presented significant deficits in attention, as well as problems in short-term memory, abstraction, and visual recognition (Riva et al., 2015b).

### Thrombotic thrombocytopenic purpura

TTP is another rare coagulation disorder of the blood-coagulation system, and is represented by small blood clots that may cause heart problems, renal failure, fever, and neurological symptoms (George, 2006). The main neurological disorders are a consequence of platelet thrombi, which may indicate infarction in many organs including the brain (Lämmle et al., 2005). Neurological symptoms include altered consciousness, confusion, seizures, and encephalopathy, which may be accompanied by neuropsychological deficits (Kennedy et al., 2009; Lewis et al., 2009).

Neurological alterations represent the hallmark of TTP's acute phase (first 6 months); however, persisting neurological symptoms may also be present in the remission phase. In a previous work, we reported that, in a small cohort of TTP patients in remission phase, 85% had a neuropsychological evaluation with an abnormal result. Among the most affected domains were attention and memory (Ferrari et al., 2015). These data were in accordance with the few other studies published on this condition (Kennedy et al., 2009; Lewis et al., 2009).

## Analysis of previous published data

Since anatomic-functional data pertaining to attention deficit in these coagulation disorders is unfortunately lacking in the literature, the aim of this work is to propose an explanatory hypothesis for attention deficit from a neuropsychological point of view, discussing the role of the frontal-subcortical circuitry, which seems to have a relevant impact on attention.

We shall consider only attention deficit, since this particular problem was experienced in both cohorts and it appeared significant. The frontal-subcortical circuitry provides a common framework for understanding the neuropsychological deficits that are associated with neurological disorders, with most of the data arising from neurodegenerative disorders and attention-deficit hyperactivity disorder (ADHD) (Durston et al., 2011).

The last 20 years have borne witness to a substantial improvement in our knowledge of the anatomy, physiology and functioning of the frontal-subcortical circuits (Bonelli and Cummings, 2007). This improvement has been accompanied by data from neuropsychological evaluations in different neurological and psychological conditions, and the results have led to the identification of the frontal-subcortical circuit as the main factor involved with neuropsychological dysfunction in the attention and executive domains (Tekin and Cummings, 2002).

From an anatomic point of view, the involvement of the frontal-subcortical circuit in executive functions seems to be associated with particular areas, such as the striatum, the globus pallidus, the substantia nigra, and the thalamus, which allow the human mind to interact adaptively with the environment (Chudasama and Robbins, 2006). Being a connector with the frontal lobe regions and the striatum, the frontal-subcortical circuit controls motor, cognitive, and behavioral abilities within the brain (Buckner, 2004). For this reason, the frontal-subcortical circuit plays a key role in attention and executive functions. While the attention function mainly pertains to alerting and orienting, “executive functions” comprise many functions, such as: perception and selection of information; processing of information in working memory; planning and decision making; and behavioral organization and adaptation. These abilities and their neuroanatomical correlates have been closely studied in neurodegenerative disorders such as Alzheimer's disease and Parkinson's disease, as well as in psychological disorders such as depression, obsessive-compulsive disorder (OCD), and ADHD (Tekin and Cummings, 2002).

We hypothesize that also in hemophilia and TTP, attentional deficits may be correlated with the involvement of the frontal-subcortical circuit. We obtained results in neuropsychological testing and in sub-domains similar to those observed in patients with dysfunction in the executive domain. Indeed, there are multiple attention systems in the brain, however we found the main problems to lie in sustained and divided attention, as reported by studies in neurodegenerative disorders (Johannsen et al., 1999; Green, 2002) and ADHD (De La Fuente et al., 2013).

Moreover, we noted major problems with attention in elderly patients in both cohorts of a hemophilia/HIV study and a TTP study. This also seems to be correlated with the results in the literature on frontal-subcortical circuitry.

In the elderly, frontal-subcortical circuitry seems to be more susceptible to white matter alterations, neuronal atrophy, and evident types of neurotransmitter depletion (Buckner, 2004). This is evident when comparing the normal aged brain and the brain of a person with Alzheimer's disease.

## Discussion

Changes in the frontal-subcortical circuitry appear to be one of the main variables causing impairments in attention and executive function (Marchand et al., 2005; Bonelli and Cummings, 2007). Such impairments affect memory, and therefore influence mental recall and controlling processes, such as memorization. These are very common problems experienced by the elderly (Buckner, 2004). A number of studies have observed age-associated attention and executive difficulties occurring in neurologically normal elderly adults (Ylikoski et al., 1995; Longstreth et al., 2002; Buckner, 2004). These results are correlated with our findings in both cohorts (TTP and hemophilia), in which older patients were shown to be more impaired than younger patients.

Data from the neuropsychological literature on aging seems to be in accordance with data from the neurophysiological literature, in which MRI studies identified that all cortical and subcortical volumes show some level of reduction with age (Buckner, 2004). Recent studies examining age-associated differences in cortical thickness report similarly widespread regional effects, with some of the more noticeable reactions observed in the frontal-subcortical regions (Salat et al., 2004). A direct correlation between frontal atrophy, attention, and executive decline was also found (Raz et al., 1998).

Patients with coagulation disorders, such as HIV + hemophilia and TTP, often report problems with attention, focus/concentration, and fatigue in their everyday life, together with other problems such as pain or mobility difficulties that are typical and well studied in chronic conditions (Schulz et al., 2013; Riva et al., 2014a). Although these problems may not be documented on routine clinical evaluations, they can be a source of serious limitation and frustration for patients with a significantly reducedhealth-related quality of life (Krasuska et al., 2012; Gringeri et al., 2013), together with considerable levels of anxiety and depression (Falter et al., 2015; Ferrari et al., 2015).

These problems appear to be distinctive and in line with diffuse frontal-subcortical circuit changes. Our hypothesis was derived from two pilot studies, which have been subject to scientific scrutiny, published, and presented at international conferences. Studies of larger patient groups are warranted in order to gain a better understanding of these preliminary observations, and to determine the anatomic-functional data characteristics that may contribute to persistent cognitive problems.

It is hoped that greater resources will be allocated and targeted toward research into the frontal-subcortical regions and their relationships with coagulation disorders in order to secure further knowledge of the neurological and neuropsychological outcomes of hemophilia and TTP.

## Author contributions

SR conceptualized the manuscript, review data literature and wrote the manuscript. SR, CF, MM review literature data and wrote the manuscript. GP conceptualized the manuscript and wrote the manuscript.

### Conflict of interest statement

The authors declare that the research was conducted in the absence of any commercial or financial relationships that could be construed as a potential conflict of interest.
